# Development and Effects of College-Based Lifestyle Modification Program for Menstrual Health of Young Adult Women with Irregular Menses: A Randomized Controlled Trial

**DOI:** 10.3390/ijerph18010233

**Published:** 2020-12-30

**Authors:** Young-Joo Park, Hyunjeong Shin, Songi Jeon, Inhae Cho, Hyun Ji Park

**Affiliations:** College of Nursing, Korea University, Seoul 02841, Korea; yjpark@korea.ac.kr (Y.-J.P.); hyunjshin@korea.ac.kr (H.S.); j50792@korea.ac.kr (S.J.); phyungi92@korea.ac.kr (H.J.P.)

**Keywords:** irregular menstruation, women, healthy lifestyle, young adult, randomized controlled trial

## Abstract

*Purpose*: This study was conducted to develop the ‘College-based Lifestyle Modification Program’ (College-based LMP) for young adult women with irregular menstruation and examine its effects after intervention. *Methods*: The College-based LMP consisted of small group education, individual physical exercise counseling/training, individual diet counseling, and feedback and support. Participants were comprised of 38 females who reported less than 10 irregular menstruations in a year and were randomly assigned to the experimental and control groups. The primary outcome variables consisted of menstrual cycle index (MCI), sex hormone binding globulin (SHBG), and androgenic profile (testosterone—T, free androgen index—FAI), while the outcome variables included premenstrual symptoms (PMS), menstrual volume, body composition parameters, glycemic parameters (fasting blood sugar—FBS, insulin, HOMA-IR), sleep duration, perceived stress, and nutrient intake.. *Results*: There were no significant differences in primary outcome variables (MCI, SHBG, T, and FAI). In the variables, there were no significant differences except for the partial domain of PMS (symptoms of depression and anxiety) and sleep duration. *Conclusions*: The study was significant in that it demonstrated the importance of lifestyle, which could provide ordinary young adult women with healthy menstruation. The College-based LMP needs to be elaborated with further studies.

## 1. Introduction

Irregular menstruation is a characterized abnormal uterine bleeding (AUB) together with unpredictable menstrual volume, and the regularity of menstruation has been taken as an indicator of women’s health [1]. A regular menstrual cycle demonstrates a normal sex hormone profile and functioning of reproductive organs, whereas irregularity may suggest a dysfunctional sex hormone profile or disorder of reproductive organs [2].

AUB appearing in the early stage of adulthood comprising the period from puberty to ages before 30 (young adult women) is involved with diverse factors; among them, ovulatory dysfunction is dominant, and the bleeding patterns can range from amenorrhea to irregular heavy menstrual bleeding [1]. Ovulatory dysfunction in young adult women is mostly a result of immaturity or temporary/chronic disturbances of the hypothalamic–pituitary–ovarian axis (HPO axis) [1]. The temporary or chronic disorder in the HPO axis is associated with androgen excess syndrome (ex. polycystic ovary syndrome; PCOS), hypothalamic dysfunction (due to eating disorders, weight loss and dieting, obesity, excessive physical exercise, poor nutrition, alcohol and drug abuse, or stress), thyroid diseases, and so on [1,3].

According to a study investigating causes about secondary amenorrhea in one hospital from 1998 to 2008, PCOS, body weight, and stress occupied the dominance of 3/4 of entire causes [4]. PCOS increases the risk of obesity, dyslipidemia, type 2 diabetes mellitus, and cardiovascular diseases in addition to the reproductive problems such as infertility resulting from ovulatory dysfunction, endometrial hyperplasia, and endometrial cancer [5,6]. The three main criteria for PCOS diagnostics are clinical/biochemical hyperandrogenism, menstrual dysfunction suggesting oligo-ovulation or anovulation, and polycystic ovary [3]. However, in 2012, the criteria of insulin resistance and metabolic syndrome were newly added to the standards, thereby enabling the presentation of four phenotypes of PCOS. The phenotypes demonstrate that PCOS is heterogeneous based on genetic background or ethnicity and can be influenced by the individual’s lifestyle [6].

The eating disorders that are frequently observable from women in puberty/early adulthood also cause menstrual disorders such as amenorrhea or irregular menstruation [7]. Women with low body weight who suffer anorexia nervosa manifest behaviors of restrictive eating and women with normal body weight suffering bulimia nervosa demonstrate compensatory behaviors of binge eating with purging [7]. As result, factors such as rapid loss of body weight, low body weight, overweight, eating behaviors, and subsequent problems related with deficiency of nutrient intake or lifestyle can be associated with menstrual disorder.

Furthermore, previous studies that have investigated the causes of irregular menstruation showed results suggesting the relationship between irregular menstruation and individual lifestyles such as body mass index (BMI), ratio of body fat, perceived stress, drinking, and smoking. For instance, in the study that analyzed the factors associated with irregular menstruation of women of the age over 19 years in the data of the Korea National Health and Nutrition Examination Survey (KNHANES) conducted from 2007 to 2014, smoking, obesity, and stress were reported as relevant factors to irregular menstruation [8]. In addition, in the study that analyzed the factors relevant to irregular menstruation from 3194 Korean women between 19–40 years in the fifth KNHANES (2010–2012), perceived stress and BMI were reported as factors significantly relevant to irregular menstruation [2].

In the study conducted with 2613 Danish women from 18 to 40 years, low level of physical activities and excessive drinking appeared related to the increase of irregular menstruation [9]. The women who exhibited behaviors of lifetime binge eating showed higher frequency of amenorrhea or oligomenorrhea than women with no binge eating behaviors [10]. Additionally, in the longitudinal study that traced menstruation conducted for one year for 54 women who restored their body weight of BMIs over 18.5 at the time of discharge from hospitals after treatment of anorexia nervosa, 35.2% were reported with resumption of menstruation; the ratio of body fat was reported as a major predictive factor of the resumption of menstruation [11]. In addition, women with insufficient sleep were at increased risk of menstrual disturbances and insulin resistance [12].

In short, previous studies have primarily suggested an approach to control individual lifestyles such as BMI, ratio of body fat, smoking/drinking, physical activities/exercises, stress, dietary uptake, and sleep to improve menstrual health for women in their puberty/early adulthood suffering from amenorrhea or oligomenorrhea.

In the meantime, intervention studies based on modified lifestyle including exercises and dietary control for women suffering irregular menstruation such as amenorrhea or oligomenorrhea were mostly conducted for obese or overweight women diagnosed with PCOS [13]. Little attention has been given to non-overweight women with PCOS [14] or for women only having problems of irregular and infrequent menstruation such as amenorrhea or secondary oligomenorrhea in healthy population. In particular, it is unknown whether modified lifestyle behaviors can lead to improved biochemical androgenic and metabolic indicators in these women with irregular and infrequent menstruation.

To fill the gaps, this study first intended for the development of the ‘College-based Lifestyle Modification Program (College-based LMP)’ for the menstrual health of young adult women, who are experiencing irregular menstruation such as amenorrhea or oligomenorrhea. Second, the developed College-based LMP was applied to young adult women experiencing irregular menstruation to verify the effects of improving menstrual health. Accordingly, the hypotheses of this study to evaluate the effects of the developed College-based LMP, were as follows:

1. There will be differences in variables of primary outcome (menstrual cycle index—MCI), sex hormone-binding globulin (SHBG), and androgenic profile) between the experimental group who were provided with College-based LMP and the control group.
2. There will be differences in the variables of outcome (premenstrual symptoms—PMS), menstrual volume, glycemic parameters, sleep duration, perceived stress and body composition parameters, and nutrients intake) between the experimental group and control group.

## 2. Methods

### 2.1. Study Design

The study intended to develop a College-based LMP for healthy menstruation of young adult women with irregular menstruation including secondary amenorrhea and oligomenorrhea, and for the verification of its effects by randomized controlled trial (RCT).

### 2.2. Setting and Samples

This study’s subjects were selected among 121 female students in the undergraduate and graduate school of K-University from a study conducted in 2017 [15]. The selection criteria were female students who reported irregular menstruation and menstruating less than 10 times in a year. Exclusion criteria were female students taking oral contraceptive pills (estrogen, progestin) or other drugs associated with menstrual disorder, or those under pertinent care.

The sample size was estimated with previous study [16]. The study was done for females with PCOS and compared its effects between the group taking metformin and the group applying a lifestyle modification program. In the previous study, the MCI changes (baseline MCI: 0.330 ± 0.194; at six months MCI: 0.706 ± 0.097) were reported from the group applying the lifestyle modification program. On this result basis, the effect size 1.0 was applied to this study. Thereby, the number of participants was calculated as 34 subjects based on an effect size 1.0, significance level (α) of 0.05, and power (1-β) of 0.80 by G*power 3.1.9.2. (Heinrich Heine University, Dusseldorf, Germany).

**Figure 1:**
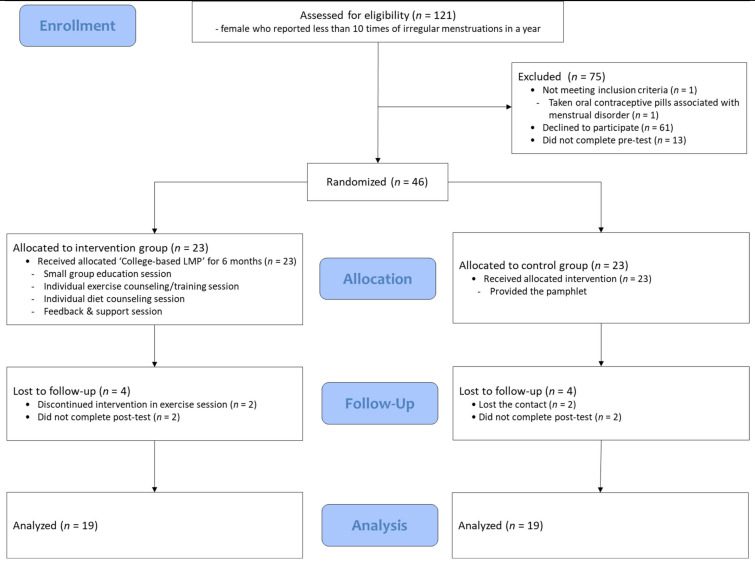
Participant flow diagram. Note. College-based LMP = College-based Lifestyle Modification Program.

However, the number of participants was determined as 46 subjects, comprising 23 as per the experimental and control group, by considering the possible drop out due to the long period (six months). The randomized assignment was done by simple randomization using the random table containing individual IDs, which were allocated on completion of pre-test conducted before the application of intervention. Intervention allocation was not concealed for participants because of the nature of the program. The final analysis included 38 participants, comprising 19 women in each group. Four participants in each group quit their participation in exercises during intervention and were absent from the post-test (Figure 1).

### 2.3. Ethical Consideration

This study was approved by the Institutional Review Board (IRB) of the university where the researcher served (IRB No. 1040548-KU-IRB-18-83-A-1). The participants, who agreed to participate in the study, were provided with an informed document about the purposes of the study, utilization of collected data, risk/benefit on participation, confidentiality of personal information, procedures for storing and destroying questionnaire/samples after experiment, and the right to withdraw from the at study anytime. Their consent on participating in the study was voluntarily signed by participants. In addition, the explanations on securing the anonymity of participants, and the usage of data restricted to purposes of research were provided with all the procedures complying with regulations of KUIRB. The participants in the control group, who were restricted to participation in the College-based LMP, were provided with a pamphlet (‘Healthy menstruation and lifestyle’) after pre-test. After post-test, they were provided with a nutrient intake evaluation and the results of the blood examination.

### 2.4. Measurements and Instruments

#### 2.4.1. Primary Outcome Variables

MCI: In this study, MCI was defined as a ratio, the number of menstruations during the experiment (six months) divided by six. A ratio close to one implies the menstruation occurs frequently.

SHBG & Androgenic Profile: The total testosterone (T) and SHBG were measured through blood examination. Thereafter, the free androgen index (FAI) was calculated by a formula consisting of T and SHBG [FAI = T (nmol/L) * 100/SHBG (nmol/L)]. Normal ranges of SHBG and T were distributed in the ranges of 32.40–128.0 nmol/L and 0.084–0.481 ng/mL, respectively; and the criteria for biochemical hyperandrogenism were either T ≥ 0.520 ng/mL or FAI ≥ 5.36 as presented in the previous study [15].

#### 2.4.2. Outcome Variables

Menstrual volume: In this study, the modified pictorial blood assessment chart (PBAC) was employed to measure the menstrual volume. PBAC was designed to appraise the loss of menstrual blood in a previous study [15,17]. It visualizes the blood clots on a pad or tampon to record the amount of blood. The scale factors for the pad comprised of ‘little’ denoted by 1 point, ‘ordinary’ by 5 points, and very large’ by 20 points, whereas the scale factors for the tampon comprised of ‘little’ denoted by 1 point, ‘ordinary’ by 5 points, and ‘very large’ by 10 points. The size of blood clots was analogized with the size of count where the ‘little’ was counted as 1 point while the ‘large’ counted as 5 points. The higher the total score implies a bigger volume of menstruation, a total score exceeding 100 points was defined as menorrhagia.

PMS: For the measurement of PMS, Daily Record of Severity of Problems (DRSP) was used [18,19]. DRSP was developed to discriminate PMS/premenstrual dysphoric disorder (PMDD) of Diagnostic Statistical Manual-IV (DSM-IV). In this study, the Criteria A of DSRP consisting of 21 questions providing 11 domains for the assessment of PMS, which was used in the previous study [15], was used as it was in the retrospective measurement on the first day of menstruation. The scores of items were distributed in the range from ‘no symptoms’ of 1 point to ‘very severe’ of 6 points; a total score of over 50 points on 21 questions in Criteria A were interpreted as experiencing PMS. In this study, Cronbach’s alpha representing internal consistency of 21 items in Criteria A was 0.93.

Glycemic parameters: The homeostatic model assessment-insulin resistance (HOMA-IR) was calculated using the formula [HOMA-IR = Insulin (μU/mL) * FBS (mg/dL)/450]. Fasting blood sugar (FBS) and level of insulin were measured through blood examination. In this study, the normal ranges of FBS and insulin were 70–110 mg/dL and 2.6–24.9 µU/mL, respectively.

Body composition parameters: Body fat, muscle mass, ratio of body fat, and body weight were measured by the body composition analyzer (InBody 230, InBody, Seoul, Korea). At pre-test, the heights of the participants were also measured using the measuring instrument of height (DS-102, Dong Sahn Jenix Co. Ltd., Seoul, Korea). According to the criteria presented by Korean Society for the Study of Obesity, we categorized BMI (kg/m^2^) less than 18.5 as underweight; 18.5–22.9 as normal; 23–24.9 as overweight; 25–29.9 as obese class I; and above 30.0 as obese class II.

Nutrients intake: A questionnaire designed to record the frequency of food intake was used for the calculation of nutrient intake. It was configured by referring to the 5th- and 7th Korea National Health and Nutrition Examination Survey (KNHANES); this was employed in a previous study [15] and applied in this study as is. It consists of 11 groups of foods (grains, beans, meats/eggs, fishes, vegetables, seaweeds, fruits, milk (products), beverage, alcoholic liquors and others), and the frequency of intake of foods was distinguished into nine categories: daily ‘3′/‘2′/‘1′ times, weekly ‘4~6′/‘2~3′/‘1′ times, monthly ‘2~3′/‘1′ times, and ‘almost no intake’. In the category of ‘fruits’, the ‘fruits of season’ was distinguished additionally, and was provided with weight as it was included in the 7th KNHANES. The standard amount of each uptake of foods was specified, where the amount of ‘little’, ‘ordinary’, and ‘plentiful’ of foods were selected by participants whose weights thereof were granted as 0.5, 1, and 2, according to the database of CAN-Pro 5.0 (for Professionals; The Korean Nutrition Society, Seoul, Korea).

Sleep duration/perceived stress: Sleep duration means the average sleeping hours per day, whereas perceived stress was defined as a degree of stress felt subjectively, which was measured based on the response to one question. The answers distributed in the range of ‘none’ of 1 point to ‘very severe’ of 6 points; the higher score point was interpreted as higher level of stress felt subjectively.

General characteristics: The questionnaire on the general characteristics of participants consisted of items on age, marital status, socioeconomic status, enrollment, and grade of university/graduate school, and the age of menarche.

### 2.5. Experiment Application and Data Collection

#### 2.5.1. Pre-Experiment and Post-Experiment Data Collection

The pre-experiment data collection consisting of questionnaires, measurement of body composition, and blood examination were carried out from 11 June to 30 June, 2018. The post-experiment data collection was performed from 26 December to 31 December 2018. The measurement of body composition and blood examination were conducted from 9 o’clock to noon, in the Health Center of K-University.

The data collection for the pre- and post-experiment was led by three researchers who were licensed nurses. The time needed for sessions of questionnaires, measurement of body composition, and blood examination was 20 to 30 min. Researchers who collected data did not become aware of which group the participants belonged to. The bloods collected from participants were refrigerated after centrifugation. For the analysis of blood, the bloods were transferred to and analyzed by the C-Medical Foundation directly in the afternoon of the day of blood collection.

#### 2.5.2. College-Based LMP for Menstrual Health

College-based LMP for menstrual health was developed by the research team based on the results of studies in the literature associated with internal- and external environmental factors (e.g., physical activity, fit body mass, diet, nutrients etc.) relevant to aspects of menstruation cycle. The College-based LMP, preliminarily developed for healthy menstruation, was modified and supplemented upon completion of validation of the program through the advice of two specialists in the disciplines of physical education and nutrition. The finally elaborated College-based LMP comprised of four sessions (small group education, individual physical exercise counseling/ training, individual diet counseling, and feedback and support). The contents in each session are as follows:

First, the small group education session provided a small group of participants with education on the knowledge of menstruation to help them understand healthy menstruation. We developed a pamphlet entitled ‘Healthy menstruation and lifestyle’ for this session. Contents of the pamphlet included: ‘Understanding of female reproductive organs’, ‘Ovulation and physiology of menstruation’, ‘What is heathy menstruation?’, ‘Issues on healthy menstruation’, ‘Health issues related with abnormal menses (particularly for irregular menses, oligomenorrhea or secondary amenorrhea)’, ‘Relationship between menstruation and lifestyle’, and ‘Lifestyle for menstrual health’.

Second, the session of ‘Individual physical exercise counseling/training’ intended for accomplishment of ‘Fit body mass’ was based on analyses of individual body composition parameters that were identified at pre-test. The physical exercise comprised of aerobic exercise using a treadmill and bicycle for 30–45 min, 3–4 times in a week, and anaerobic exercise to reinforce muscle power, which were selected by the preference of individuals. The fitness center, located around the university, was provided as a place for exercise; the individuals participated in exercises at their own preferred times.

Third, in the ‘Individual diet counseling session’, the participants were provided with counselling on the intake of optimal calorie and balanced nutrition. This was based on the result of analyses on the body composition parameters and nutrient intake of participants identified through pre-test in a previous study [15]. We developed the ‘Diet counselling record’ by individual. The title of the developed ‘Diet counselling record’ was ‘What is the desirable nutrition for myself?’ and was comprised of the following items: ‘How is my body composition and BMI?’, ‘The intake and evaluation on nutrients for myself (29 nutrients: calorie, macronutrients, fat-soluble/water-soluble vitamins, macro/micro minerals)’, and ‘Recommended dietary intake (RDI)’.

Fourth, the feedback and support session provided the participants with rewards (complimentary mobile text and coupons) of positive feedback in cases of accomplishment of individual objectives by the degree of participation in individual exercises every month.

#### 2.5.3. Experimental Application

The experimental group was provided with the applications of the following components in College-based LMP to improve the health of menstruation. In the small group education session, the participants were supposed to participate in a session at the available times of 10 a.m., 2 p.m., and 4 p.m. among the four days of 25, 26, 28, and 29 June 2019 using the ‘Healthy menstruation and lifestyle’ pamphlet. The number of participants in the small group was 2–5 people and the session went for 40–50 min. A lecture room in K-University was provided for the session. The session of individual physical exercise counseling/training was carried out in the adjacent fitness center 3–4 times a week, with a session going for 30–45 min for six months, thus supporting the sustainable participation in physical exercises of the participants through contract. The individual participant objectives about physical exercise were shared with specialists at the fitness center to be available for participants on request. The specialists advised and supervised the participants during exercise on an individual basis. The participants who participated in exercises 3–4 times every week were compensated with a monthly reward.

The session of individual diet counseling was performed individually based on the ‘Diet counselling record’ upon completion of the education of the small group. The session required 10–20 min of time and was conducted by the research director.

The participants in the control group were provided with the pamphlet, ‘Healthy menstruation and lifestyle’, which was used in the small group education session through online services after completion of the pre-test.

### 2.6. Data Analysis

The collected data were analyzed by the pc-SAS Program (Version 9.2, SAS Institute, Cary, NC, USA), while the analysis of nutrient intake was undertaken by the CAN-Pro 5.0 (for Professionals). Analysis followed per protocol analysis.

The assumption of the normal distribution of variables were tested by the Shapiro–Wilks test, and for the cases that failed to fulfill the assumption, the nonparametric test was carried out. To identify the pre- and post-test intergroup- and intragroup differences in MCI, SHBG, androgenic profile, glycemic parameters, sleep duration, perceived stress, body composition parameters, and general characteristics, the Wilcoxon rank sum test was used. The t-test and ANCOVA were carried out for the analysis of pre- and post-test intragroup- and intergroup differences in PMS between the experimental- and control group. To attain the descriptions and distributive characteristics of MCI, SHBG, androgenic profile, PMS, menstrual volume, sleep duration, perceived stress, glycemic parameters, body composition parameters, nutrient intake, and general characteristics of participants in the experimental and control group, descriptive statistics such as frequency, percentage, mean, and standard deviation of variables were used. For the analysis on the difference between the experimental and control group, the t-test, Wilcoxon rank sum test, and Fisher-exact test were conducted.

## 3. Results

### 3.1. Similarity between Experimental and Control Group

No statistically significant differences were found at baseline scores between the experimental- and control group in variables as follows: (a) general characteristics (age, socioeconomic level, marital status, occupation, age of menarche); (b) variables of primary outcome—MCI, SHBG, androgenic profile (T, FAI); and (c) variables of outcome—PMS, menstrual volume, glycemic parameters (FBS, Insulin, HOMA-IR), sleep duration, perceived stress, body composition parameter (body fat mass, muscle mass, ratio of body fat, BMI), nutrients intake; (Table 1, Table 2, Table 3, Table 4 and Table 5).

### 3.2. Primary Outcome Analysis

The results of the primary outcome analysis are as summarized in Table 2.

The score of MCI increased 0.49 to 0.65 in the experimental group, and 0.54 to 0.74 in control group, respectively, after intervention (after six months). MCI score of the control group appeared lower than the experimental group in post-test, however, it showed no statistical significance (Z = −1.07, *p* = 0.285).

The level of SHBG increased 63.05 nmol/L to 80.67 nmol/L in the experimental group, and 77.04 nmol/L to 82.45 nmol/L in the control group. The level of increase regarding SHBG appeared larger in the experimental group than in the control group. However, the post measurement of SHBG exhibited no significant differences between the two groups (Z = 0.00, *p* < 0.999).

Among the androgenic profiles, the level of T in the experimental group varied from 0.38 ng/mL to 0.40 ng/mL, whereas that of the control group changed from 0.44 ng/mL to 0.43 ng/mL; the post measurement of T of the experimental group appeared lower than that of the control group, however, no significant differences were found (Z = 0.07, *p* = 0.942). Regarding FAI, the score of FAI in the experimental group exhibited a slight increase from 2.54 to 2.70, while the score in the control group dropped slightly from 3.29 to 3.13; the post score of FAI in the experimental group appeared slightly lower than that of the control group, however, it showed no significant differences (Z = −0.06, *p* = 0.953).

### 3.3. Outcome Analysis

Regarding PMS, the participants in the experimental group showed a decrease from 53.11 to 44.32, whereas the participants in the control group exhibited a decrease from 57.21 to 53.67; the level of decrease appeared greater in the experimental group, however, no significant differences between the two groups were found from the analysis of covariance taken pre-scores as covariate (F = 1.81, *p* = 0.188). Among the 11 domains of PMS, the depression scores in the experimental group showed a decrease in score from 6.11 to 4.63, whereas the score in the control group showed slight increase from 7.26 to 7.28; the difference between scores of the two groups appeared statistically significant in the analysis of covariance taken pre-scores as covariate (F = 4.95, *p* = 0.033). In the domain of anxiety, both the experimental- and control group commonly showed decreases from 3.26 to 1.95 (experimental group) and 3.42 to 3.06 (control group); the level of decrease in the experimental group appeared greater than that in the control group, and the results in the analysis of covariance taken pre-scores as covariate showed statistical significance in the difference between scores (F = 5.82, *p* = 0.021). The rest domains showed no statistically significant differences between the two groups (Table 3).

Regarding the menstrual volume, the participants in the experimental group showed a decrease from 109.11 to 106.37 while the participants in the control group showed an increase from 108.53 to 117.42; however, no statistically significant differences in menstrual volumes before and after the experiment between two groups were found (z = 0.88, *p* = 0.381) (Table 4).

Regarding the glycemic parameters, the post-test FBS (z = −0.66, *p* = 0.511), insulin (z = 0.26, *p* = 0.793), and HOMA-IR (z = 0.15, *p* = 0.884) all showed no statistically significant differences between the two groups. The analysis on the differences between pre- and post-test scores showed no statistically significant differences in each group except the FBS of the control group (Table 4).

The experimental group’s sleep duration increased from 6.21 h to 6.63 h, while it decreased from 6.37 h to 5.74 in the control group; the difference in sleep duration between the two groups was statistically significant (z = 2.23, *p* = 0.026). Perceived stress of participants in the experimental group decreased from 3.58 to 3.32 and increased from 3.89 to 4.00 in the control group. However, the difference between the groups was statistically insignificant (z = −1.77, *p* = 0.078) (Table 4).

Regarding body composition parameters, the body fat mass (z = 1.22, *p* = 0.224), muscle mass (z = 0.49, *p* = 0.627), BMI (z = 0.93, *p* = 0.354), and ratio of body fat (z = 0.79, *p* = 0.430) of the participants showed no statistically significant differences between the two groups after intervention (Table 4).

In the analysis of pre- and post-test scores of nutrient intake, all 16 nutrients showed no statistically significant differences between the two groups after intervention, and between pre- and post-test scores in each group (Table 5).

## 4. Discussion

In the study, the hypotheses, which stated that there will be differences in variables of primary outcome such as MCI, SHBG, T, and FAI between participants of the experimental group provided with College-based LMP and the control group upon completion of the trial after six months, were rejected with insignificant differences. That is, the positive effect of College-based LMP, which was defined as MCI, SHBG, T, and FAI to represent the improvement in menstrual health, were not identified.

These findings differ from those reported in previous studies. For example, in a study that carried out dietary control and physical exercises for four months for female of severe obesity suffering from PCOS, the results showed improvement in the level of total testosterone and SHBG [20]. In addition, there was the meta-analysis that reported the effect of lifestyle intervention including exercises and dietary control in overweight/obese females suffering from PCOS and significant positive effects regarding FSH, SHBG, total T, androstenedione, FAI, and Ferriman-Gallwey Score were shown. The subjects of the seven studies included in the analysis were also overweight/obese PCOS women with an average BMI of 26.8 to 40.4 [21]. In contrast, few studies have ever been carried out with women of normal body weight but suffering PCOS. In prior studies, the positive results of decreasing menstruation period from 46.1 days to 27.3 days from females in the experimental group were reported by the structured exercise that lasted for 8-weeks and applied to females of normal body weight suffering PCOS [14].

However, the participants in the present study were young adult women who reported experiences of irregular menstruation and oligomenorrhea less than 10 times a year with BMIs in the normal range; a mean value of 21.25 for the experimental group and 21.56 for the control group, distributed within the range 16.4–28.1. In this study, the number of subjects suspected to be suffering from PCOS was five in the experimental group and six in the control group; this was estimated based on the criteria (T > 0.520 ng/mL or FAI > 5.36) employed in previous study [15]; among them, two in the experimental group and three in the control group had overweight/obesity (BMI ≥ 23). Thus, there are limitations in comparing the results of the present study with the results of lifestyle modification for women with PCOS in the previous studies cited above.

Nonetheless, the results of analysis on the changes in MCI and SHBG, before and after six months of the trial between the experimental- and control group, showed a common increase of MCI from 0.49 to 0.65 and from 0.54 to 0.74, respectively. Regarding SHBG, the two groups appeared with a common increase of SHBG from 63.05 nmol/L to 80.67 nmol/L in the experimental group and from 77.04 nmol/L to 82.45 nmol/L in the control group. The College-based LMP in this study consisted of small group education participation, exercise, nutrition counseling, and feedback and support. Of these, the educational materials used for small group education were provided to the experimental group at the time of participation in education, and to the control group by email. This may have a positive effect of improving menstrual health even though the control group did not directly participate in small group education, and this may cause an effect on the change in the positive direction of MCI or SHBG. However, in this study, individual nutrition counseling, which is a component of the program, was provided to the experimental group based on the individual dietary intake analysis data, but was not directly intervened by actual nutrition and dietary management. Moreover, since the subjects of this study were undergraduate or graduate students in early adulthood, it may be limited to apply them in conjunction with nutrition and dietary management in their lives. Therefore, further research is required to include specific methods and strategies to ensure that each component of the college-based LMP can be applied to real life.

This study used following variables of outcome to identify effects of College-based LMP, PMS, sleep duration, perceived stress, menstrual volume, glycemic parameter (FBS, Insulin, HOMA-IR), body composition parameters, and nutrient intake. Accordingly, we made the hypothesis that stated that there were differences in these variables between the experimental group who were provided with the College-based LMP and the control group after intervention (after six months). However, the differences appeared insignificant except for the partial domain of premenstrual symptoms (depression, anxiety) and sleep duration. Thus, these hypotheses, except for parts of premenstrual symptoms (depression, anxiety) and sleep duration, were rejected.

Regarding PMS, the score of the experimental group decreased from 53.11 to 44.32 while the score of the control group decreased from 57.21 to 53.67, showing a bigger decrease in the score of the experimental group, but indicating statistically insignificant variation. In the analysis of each domain of PMS, the experimental group showed significant decrease in two domains, the depression and anxiety, among 11 domains than those of the control group. This seems ascribable to the results of the composition of College-based LMP that consisted of exercises and dietary control intended for the improvement of menstrual health.

Sleep duration increased in the experimental group after intervention, whereas it appeared to decrease in the control group with the difference indicating statistical significance. One recent study reported that women who reported less than 6 h of sleep had significantly higher odds (OR = 2.1) of an abnormal menstrual cycle length (short or long), a significantly higher mean BMI, fasting insulin levels, and HOMA-IR than women with six or more hours of sleep [12]. In this regard, the increase in MCI in the experimental group and the control group after the experiment shown in this study may potentially suggest the relationship between sleep duration and MCI. Actually, in the experimental group, the association between MCI and sleep duration was significant (r = 0.470, *p* = 0.042). The perceived stress decreased in the experimental group, while it increased in the control group, but it was statistically insignificant (t = −1.77, *p* = 0.078).

Regarding menstrual volume, the experimental group manifested a decrease from 109.11 to 106.37, while it appeared to increase from 108.53 to 117.42 in the control group with the difference showing no statistical significance. The frequency of menorrhagia of young adult women participated in this study appeared higher than 21.8% of Turkish female university students who participated in the study employing the PBAC, which was used in the present study [22]. Contrary to the cited study that studied ordinary population as subjects, in this study, the subjects consisted of women who reported irregular menstruation in the aspect of oligomenorrhea experiencing less than 10 times a year among an ordinary population of females in the early stage of adulthood; thus, the subjects participated in the present study may represent potential possibilities of unpredictable menorrhagia together with irregular menstruation.

Variables of glycemic parameters (FBS, insulin, HOMA-IR) and parameters of body composition (body fat mass, ratio of body fat, BMI, muscle mass) all showed differences of no statistical significance between two group participants in the present study. In the meta-analysis and systematic review regarding the effects of lifestyle intervention on the body composition of overweight/obesity women with PCOS, the exercises alone or exercises with dietary intervention, that is, the lifestyle intervention including exercises, had positive effects on parameters of body composition than sedentary control, placebo, diet only or usual care including metformin [23]. In addition, lifestyle intervention was reported from a systematic review on non-drug intervention that exhibited the effect of improvement in androgenic syndrome such as hirsutism in glycemic outcomes and in decrease of BMI [24].

In contrary to studies cited above, the participants in this study consisted of women of low/normal weight (experimental group: 73.7%, control group: 84.2%). Therefore, the variables of body composition or glycemic parameters representing changes between pre- and post-test should be considered that the sensitivity of variables may not be sufficient enough to detect distinguishable changes.

Nonetheless, results suggesting a relationship between oligomenorrhea and metabolic syndrome of females with normal body weight, have been reported from a study conducted recently. The study was conducted on 1174 females from the ages of 19 years to 39 years experiencing irregular menstruation, where the females suffering from severe oligomenorrhea (severe OM; menstrual cycle length >60 days) and mild oligomenorrhea (mild OM; menstrual cycle length 40–60 days) were compared to each other. The subjects of severe OM appeared with the odds ratio of metabolic syndrome risk twice higher than subjects of mild OM [25].

Regarding the analysis of nutrient intake of the 16 nutrients (calorie, carbohydrate, fat, protein, fiber, vitamin D, folic acid, calcium, phosphorus, sodium, magnesium, iron, selenium, omega-3, omega-6, chromium picolinate), no statistically significant difference was observed between the two groups after intervention. According to studies that reported the relationship between irregular menstruation and nutrient intake, the low level of 25-hydroxyvitamin D was found to be related to irregular menstruation; this suggests that vitamin D is involved with anti-Mullerian hormone, insulin, and androgens that may influence regularity of the menstrual cycle [26]. The administration of omega-3 supplementation to overweight/obesity women with irregular menstruation, suffering PCOS, resulted in a significant decrease of T. After trial, 47.2% of women that returned to a regular menstrual cycle in the group administered with omega-3 was higher than 22.9% in the group administered with the placebo [27]. The administration of folic acid to female subjects of BMI over 25 and suffering PCOS, resulted in the effect of significant decrease in plasma homocysteine, HOMA-IR score, and total cholesterol/HDL-cholesterol ratio [28]. Thus, for women with PCOS, a carefully prepared low-calorie diet to keep healthy weight or for weight loss for improvement of insulin resistance and metabolic- and reproductive functions, and restriction on the intake of simple sugar or refined carbohydrate, foods of low glycemic index, saturated trans-fatty acid, and taking care of deficiency in vitamin D, chromium, and Omega-3, were suggested [29]. Most studies cited above were conducted with females suffering PCOS, raising limitations in comparison with the subjects that participated in the present study. The insignificant differences of nutrient intake in the present study can be attributed to the intervention on personal nutrition counseling based on the analysis of nutrients and on personal BMIs in our program, rather than direct intervention on individual daily dietary intake; thus, this result may reflect limitations in the application of the intervention to the actual daily lives of young adult women who might have encountered difficulties in applying the intervention to day after day.

In summary, the College-based LMP showed positive effects of improvement in variables of partial domain of PMS (depression and anxiety) and sleep duration, but did not render the MCI, SHBG, T, and FAI. This study was significant in that it confirmed the effect of healthy lifestyles on menstrual health in young adult women with irregular menstruation such as oligomenorrhea and secondary amenorrhea.

There are several limitations in this study. First, factors of organic diseases (ex. Cushing’s syndrome etc.) among diverse causes of irregular menstruation such as oligomenorrhea or amenorrhea were not distinguished. Second, there is a limitation in generalizing to other women due to the results on the small number of women. Finally, the intervention time for six months might not be enough for women with healthy BMI and no previous diagnosis of PCOS. Thus, further studies are suggested to secure a larger population of subjects based on different BMIs together with supplementation to the limitations.

## 5. Conclusions

In the present study, the College-based LMP was developed to improve the menstrual health of young adult women who experienced irregular menstruation less than ten times a year, and its effects were examined through RCT. The College-based LMP consisted of the education in small groups, individual counseling on exercises and training, and individual counselling on diet and feedback and support. Consequently, the College-based LMP showed positive effects of the alleviation of depression and anxiety, which are partial domains of PMS for women experiencing irregular menstruation, and improvement of sleep duration. However, the positive effects of the College-based LMP were not found for MCI, SHBG, T, FAI, overall PMS, menstrual volume, body composition parameters, and nutrient intake. Further studies are suggested to repeat this study with supplementation to the limitations of this study.

## Figures and Tables

**Table 1:** General characteristics in the experimental and control groups at baseline (*n* = 38).

Variables		Experimental Group (*n* = 19)		Control Group (*n* = 19)		X^2^ or Z (*p*)
		Mean ± SD (Range)	*n* (%)	Mean ± SD (Range)	*n* (%)	
Age (year)		22.37 ± 2.50		21.74 ± 2.51		0.84 (0.401) ^b^
Socioeconomic status						
	High		3 (15.8)		3 (15.8)	0.00 (<0.999) ^a^
	Middle		15 (79.0)		15 (79.0)	
	Low		1 (5.3)		1 (5.3)	
Educational status						
	Undergraduate student		16 (84.2)		16 (84.2)	0.00 (<0.999) ^a^
	Graduate student		3 (15.8)		3 (15.8)	
Menarche age (yr)		12.45 ± 1.44		12.97 ± 1.99		−0.49 (0.622) ^b^
		(11.0–17.0)		(11.0–17.0)		

^a^*p*-value by fisher exact test; ^b^*p*-value by wilcoxon rank sum test.

**Table 2:** Comparison of primary outcome variables in the experimental and control groups (*n* = 38).

Variables		Experimental Group (*n* = 19)	Control Group (*n* = 19)	Z (*p*)
		Mean ± SD	Mean ± SD	
MCI				
	Pre-test	0.49 ± 0.23	0.54 ± 0.23	−0.64 (0.519) ^a^
	Post-test	0.65 ± 0.25	0.74 ± 0.26	−1.07 (0.285) ^a^
SHBG				
	Pre-test	63.05 ± 25.27	77.04 ± 58.86	−0.26 (0.793)
	Post-test	80.67 ± 60.22	82.45 ± 58.24	0.00 (<0.999)
Androgenic profile				
T (ng/mL)	Pre-test	0.38 ± 0.19	0.44 ± 0.29	−0.69 (0.493)
	Post-test	0.40 ± 0.20	0.43 ± 0.23	0.07 (0.942)
FAI	Pre-test	2.54 ± 1.92	3.29 ± 3.05	−0.32 (0.748)
	Post-test	2.70 ± 2.37	3.13 ± 3.18	−0.06 (0.953)

^a^*p*-value by wilcoxon rank sum test; FAI = Free androgen index; MCI = Menstrual cycle index; SHBG = Sex hormone binding globulin; T = Testosterone.

**Table 3:** Comparison of premenstrual symptoms in the experimental and control groups (*n* = 38).

Categories (No of Items)		Experimental Group (*n* = 19)	Control Group (*n* = 19)	t (*p*)	F (*p*) ^a^
		Mean ± SD	Mean ± SD		
Felt depressed/hopeless/worthless (3)	Pre-test	6.11 ± 3.03	7.26 ± 4.01	−1.00 (0.322)	4.95 (0.033)
	Post-test	4.63 ± 1.57	7.28 ± 4.14	−2.54 (0.019)	
Anxious, tense, or on edge (1)	Pre-test	3.26 ± 1.59	3.42 ± 1.71	−0.29 (0.770)	5.82 (0.021)
	Post-test	1.95 ± 1.18	3.06 ± 1.59	−2.42 (0.021)	
Mood swings/Sensitive to rejection (2)	Pre-test	5.05 ± 2.82	6.05 ± 3.39	−0.99 (0.329)	1.30 (0.262)
	Post-test	3.74 ± 2.10	5.11 ± 2.95	−1.64 (0.110)	
Felt Angry, irritable/Conflicts or problems with people (2)	Pre-test	4.58 ± 1.92	5.53 ± 2.84	−1.21 (0.236)	0.03 (0.861)
	Post-test	4.37 ± 2.14	5.00 ± 2.87	−0.76 (0.227)	
Less interest in usual activity (1)	Pre-test	3.05 ± 1.58	2.63 ± 1.61	0.81 (0.421)	1.49 (0.231)
	Post-test	2.68 ± 1.57	3.00 ± 1.57	−0.61 (0.544)	
Difficulty concentrating (1)	Pre-test	3.11 ± 1.68	2.53 ± 1.39	1.16 (0.255)	0.44 (0.512)
	Post-test	2.74 ± 1.52	2.72 ± 1.49	0.03 (0.977)	
Lethargic, tired, fatigued or lack of energy (1)	Pre-test	3.58 ± 1.71	3.68 ± 1.49	−0.20 (0.841)	1.05 (0.312)
	Post-test	3.16 ± 1.39	3.61 ± 1.46	−0.97 (0.339)	
Increased appetite/Cravings for specific foods (2)	Pre-test	5.42 ± 2.85	5.53 ± 2.87	−0.11 (0.910)	0.80 (0.376)
	Post-test	5.16 ± 2.59	4.39 ± 2.87	0.86 (0.398)	
Slept more/Trouble sleeping (2)	Pre-test	4.84 ± 2.61	5.42 ± 3.24	−0.61 (0.548)	0.63 (0.433)
	Post-test	4.42 ± 2.55	5.17 ± 2.23	−0.95 (0.351)	
Overwhelmed, cannot cope/Out of control (2)	Pre-test	3.11 ± 2.05	3.47 ± 2.46	−0.50 (0.619)	1.66 (0.207)
	Post-test	2.58 ± 1.17	3.50 ± 2.62	−1.37 (0.184)	
Physical symptoms (4)	Pre-test	11.16 ± 4.25	11.68 ± 4.24	−0.38 (0.705)	1.94 (0.172)
	Post-test	8.89 ± 4.08	10.83 ± 4.34	−1.40 (0.170)	
Total (21)	Pre-test	53.11 ± 19.03	57.21 ± 20.99	−0.63 (0.532)	1.81 (0.188)
	Post-test	44.32 ± 16.83	53.67 ± 21.24	−1.49 (0.146)	

^a^*p*-value by analysis of covariance (ANCOVA).

**Table 4:** Comparison of body composition parameters, glycemic parameters, perceived stress, sleeping duration, and menstrual volume in the experimental and control groups (*n* = 38).

Variables		Experimental Group (*n* = 19)	Control Group (*n* = 19)	X^2^ or Z (*p*)
		Mean ± SD	Mean ± SD	
Body composition parameters				
Body fat mass (kg)	Pre-test	17.44 ± 5.24	16.24 ± 5.52	1.18 (0.237)
	Post-test	18.32 ± 4.88	17.25 ± 5.92	1.22 (0.224)
Muscle mass (kg)	Pre-test	20.89 ± 1.83	20.32 ± 2.22	0.89 (0.373)
	Post-test	20.80 ± 1.82	20.38 ± 2.12	0.49 (0.627)
BMI (kg/m^2^)	Pre-test	21.25 ± 2.87	20.67 ± 2.41	0.95 (0.342)
	Post-test	21.56 ± 2.81	21.11 ± 2.72	0.93 (0.354)
Ratio of body fat (%)	Pre-test	30.49 ± 5.73	29.61 ± 6.94	0.73 (0.465)
	Post-test	31.75 ± 5.60	30.75 ± 7.21	0.79 (0.430)
Glycemic parameters				
FBS (mg/dL)	Pre-test	89.95 ± 7.28	89.74 ± 8.19	0.29 (0.770)
	Post-test	90.05 ± 8.51	92.68 ± 6.36	−0.66 (0.511)
Insulin (μU/mL)	Pre-test	7.50 ± 6.35	6.83 ± 4.84	0.23 (0.815)
	Post-test	7.91 ± 5.47	7.78 ± 5.40	0.26 (0.793)
HOMA-IR	Pre-test	1.72 ± 1.64	1.56 ± 1.21	0.20 (0.838)
	Post-test	1.77 ± 1.26	1.83 ± 1.34	0.15 (0.884)
Perceived stress				
	Pre-test	3.58 ± 1.07	3.89 ± 0.73	−0.87 (0.386)
	Post-test	3.32 ± 1.00	4.00 ± 1.41	−1.77 (0.078)
Sleep duration (hours)				
	Pre-test	6.21 ± 1.07	6.37 ± 1.34	−0.07 (0.941)
	Post-test	6.63 ± 1.05	5.74 ± 1.38	2.23 (0.026)
Menstrual volume				
	Pre-test	109.11 ± 81.83	108.53 ± 111.21	0.42 (0.672) ^a^
	Post-test	106.37 ± 68.67	117.42 ± 171.33	0.88 (0.381) ^a^

^a^*p*-value by wilcoxon rank sum test; BMI = Body mass index; FBS = Fasting blood sugar; HOMA-IR = Homeostasis model assessment—insulin resistance.

**Table 5:** Comparison of nutrients in the experimental and control groups (*n* = 38).

Nutrients		Experimental Group (*n* = 19)	Control Group (*n* = 19)	Z (*p*)
		Mean ± SD	Mean ± SD	
Calorie (kcal)	Pre-test	1553.77 ± 636.68	1591.37 ± 614.67	0.90 (0.365)
	Post-test	1378.45 ± 576.73	1456.64 ± 780.77	1.26 (0.209)
Carbohydrate (gm)	Pre-test	231.42 ± 100.95	242.49 ± 105.08	0.58 (0.559)
	Post-test	214.81 ± 104.75	211.17 ± 114.76	1.11 (0.267)
Fat (gm)	Pre-test	41.36 ± 24.07	40.19 ± 17.30	0.64 (0.521)
	Post-test	34.11 ± 13.46	41.83 ± 26.98	0.38 (0.704)
Protein (gm)	Pre-test	61.93 ± 29.23	61.78 ± 28.63	1.08 (0.280)
	Post-test	50.40 ± 20.30	58.50 ± 38.11	1.11 (0.267)
Fiber (gm)	Pre-test	20.34 ± 14.74	19.23 ± 14.56	1.11 (0.267)
	Post-test	13.60 ± 6.18	17.69 ± 11.64	0.47 (0.640)
Vitamin D (μg)	Pre-test	4.05 ± 2.99	4.40 ± 3.45	1.28 (0.199)
	Post-test	2.79 ± 1.73	2.84 ± 1.54	0.99 (0.321)
Folic acid (μg)	Pre-test	483.46 ± 301.23	463.17 ± 313.81	1.37 (0.170)
	Post-test	322.75 ± 102.26	417.24 ± 280.71	0.53 (0.599)
Calcium (mg)	Pre-test	582.71 ± 405.53	562.09 ± 430.70	2.01 (0.044)
	Post-test	345.41 ± 190.38	373.56 ± 279.24	1.51 (0.129)
Phosphorus (mg)	Pre-test	1044.16 ± 569.62	1017.85 ± 567.26	1.25 (0.217)
	Post-test	784.37 ± 321.77	861.81 ± 537.71	1.26 (0.209)
Sodium (mg)	Pre-test	1614.34 ± 868.01	1577.39 ± 688.19	0.15 (0.884)
	Post-test	1408.73 ± 617.77	1501.22 ± 921.96	0.64 (0.521)
Magnesium (mg)	Pre-test	95.95 ± 66.18	98.50 ± 76.57	1.28 (0.199)
	Post-test	64.25 ± 36.85	91.10 ± 60.95	0.09 (0.930)
Iron (mg)	Pre-test	13.71 ± 8.31	13.20 ± 7.90	1.28 (0.199)
	Post-test	10.02± 4.08	11.64 ± 6.87	1.14 (0.255)
Selenium (μg)	Pre-test	65.79 ± 51.14	61.90 ± 41.92	1.05 (0.293)
	Post-test	44.47 ± 21.54	52.07 ± 29.97	0.70 (0.484)
Omega-3	Pre-test	0.56 ± 0.46	0.76 ± 0.87	−0.38 (0.704)
	Post-test	0.75 ± 0.79	0.85 ± 0.93	−0.50 (0.620)
Omega-6	Pre-test	3.33 ± 2.86	3.90 ± 3.00	−0.35 (0.726)
	Post-test	4.49 ± 5.18	3.97 ± 4.58	0.29 (0.770)
Chromium picolinate (μg)	Pre-test	3.91 ± 2.93	5.10 ± 3.26	0.56 (0.579)
	Post-test	4.40 ± 3.24	3.40 ± 2.67	1.71 (0.088)

## Data Availability

The data presented in this study are available on request from the corresponding author. The data are not publicly available due to privacy.

## Trial Registration

Clinical Research Information Service (CRiS) number (KCT0005538).
